# Supplementary material for: Gramiketides, Novel Polyketide Derivatives of Fusarium graminearum, Are Produced during the Infection of Wheat
Source: J Fungi (Basel). 2022 Sep 28;8(10):1030. doi: 10.3390/jof8101030 (PMC9605136; doi:10.3390/jof8101030)
Supplement: Supplementary file 1 [file jof-08-01030-s001.zip › jof-1915903-supplementary.pdf]

Journal of *Fungi*

Supporting Material

Gramiketides, Novel Polyketide Derivatives of *Fusarium Graminearum*, are Produced during the Infection of Wheat

Bernhard Seidl [1], Katrin Rehak [2], Christoph Bueschl [1], Alexandra Parich [1], Raveevatoo Buathong [3], Bernhard Wolf [1], Maria Doppler [1,4], Rudolf Mitterbauer [2], Gerhard Adam [2], Netnapis Khewkhom [3,*], Gerlinde Wiesenberger [2,*] and Rainer Schuhmacher [1,*]

[1] Department of Agrobiotechnology IFA-Tulln, Institute of Bioanalytics and Agro-Metabolomics, University of Natural Resources and Life Sciences Vienna (BOKU), Konrad-Lorenz-Str. 20, 3430 Tulln, Austria
[2] Department of Applied Genetics and Cell Biology, Institute of Microbial Genetics, University of Natural Resources and Life Sciences Vienna (BOKU), Konrad-Lorenz-Str. 24, 3430 Tulln, Austria
[3] Department of Plant Pathology, Faculty of Agriculture, Kasetsart University, Ngamwongwan Road, Lat Yao, Chatuchak, Bangkok 10900, Thailand
[4] Core Facility Bioactive Molecules: Screening and Analysis, University of Natural Resources and Life Sciences Vienna (BOKU), Konrad-Lorenz-Str. 20, 3430 Tulln, Austria
* Correspondence: agrnpk@ku.ac.th (N.K.); gerlinde.wiesenberger@boku.ac.at (G.W.); rainer.schuhmacher@boku.ac.at (R.S.)

Table S1. Primers used in this study.

Primer	Designation	Sequence 5′-3′	Plasmid
4232	*PKS15*Dup_SfiI-fw	TAATGGCCGCATAGGCCTGAGTCGTACCTCCATTG TAGC	pKR3
4233	*PKS15*Dup_SpeI-rv	AAACTAGTTCTGATGATGATATGTAGATACTG	
4234	*PKS15*+6474-fw	GATGACCGAACATATGCACTGG	pKR9
4235	*PKS15*+6979-rv	CTAAGCGCTATCTTAGTATCTGC	
4236	*PKS15*-601-fw	GTGATGTACTGACTCGGATC	PCR-c
4237	*PKS15*+237-rv	GATCATGTATTCTGAGAAATGAG	PCR-c
3151	pII99, nptII into pUG6 rv, SacI	TATGAGCTCTTCATGCCAGTTGTTCCCAG	pPS28
3152	pII99, nptII into pUG6 fw, XbaI	TATTCTAGATCTTATCGAGATCCTGAACAC	
3200	*KMT6* downstream, fw NdeI	ATACATATGGGTAGACACGGTTCACGCT	pRS105
3201	*KMT6* downstream, rv HindIII	TTTGGGCAGAGAAGCTTGAAT	
3202	*KMT6* upstream, fw EcoRV	ATAGATATCTGGCTGCGGCTAATTGATGG	pRS106
3204	*KMT6* upstream rv SpeI	TATACTAGTCCTAAACTCCATGACCGACAG	
3273	Kmt6d_seq_fw	CCTCCACCATGCTTAGCCTG	Seq.
3274	Kmt6d_seq_rv	GGATATTGGCCAGCTCACTG	Seq.
4786	*PKS15*_mutation_fw	CGTTAAGCTTCTGGAAGACGTGTAC	pKR92
4787	*PKS15*_mutation_rv	TACACGTCTTCCAGAAGCTTAACGGCGGCT	
4782	F6_down_fw	CCACTCAGGCCCGTCTAGAGTTTGCTCAAATC	pKR92
4783	F6_down_rv	TTCTATCTGTTTCGACGTTCCTCAGGATCCTTCTAG TCTCTGAAAC	

Numbers according to primer collection (Adam Lab). “Seq.”: primer used for sequencing. “PCR-c”: flankingprimer used for PCR check of correct integration.

Table S2. Plasmids generated in this study.

Plasmid	Construction
pKR3	*PKS15* upstream region PCR fragment (primers 4232 +4233)/Sfi+SpeI into pKT248
pKR9	*PKS15* downstream region PCR fragment (primers 4234 +4235)/HindIII+SalI into pKT248
pPS28	PCR product (primers 3151+3152)/XbaI+SacI, cloned into pUG6/ XbaI+SacI,

	loxP-(P*trpC*-*nptII*-T*trpC*)-*loxP*
pRS105	Downstream *KMT6* (PCR primers 3200+3201) into pPS28/NdeI+HindIII
pRS106	Upstream *KMT6* (PCR primers 3202+3204) into pPS28/ EcoRV+SpeI
pRS107	Upsteam and downstream *KMT6* combined in pPS28
pKR92	Gibson assembly / *KMT6* flanks and ORF with additional silent HindIII

Table S3. ddMS/MS acquisition inclusion list of *m*/*z* values of theoretical X-n isotopologs of the two gramiketide $[M+H]^+$ ions.

Mass [m/z]	Polarity	Start [min]	End [min]	Comment
521.31089	Positive	24.12	26.12	gramiketide A ^{12}C
550.40819	Positive	24.12	26.12	gramiketide A ^{13}C
548.40148	Positive	24.12	26.12	gramiketide A ^{13}C X-2
546.39477	Positive	24.12	26.12	gramiketide A ^{13}C X-4
544.38806	Positive	24.12	26.12	gramiketide A ^{13}C X-6
542.38135	Positive	24.12	26.12	gramiketide A ^{13}C X-8
540.37464	Positive	24.12	26.12	gramiketide A ^{13}C X-10
538.36793	Positive	24.12	26.12	gramiketide A ^{13}C X-12
536.36122	Positive	24.12	26.12	gramiketide A ^{13}C X-14
503.30033	Positive	27.47	29.47	gramiketide B ^{12}C
532.39762	Positive	27.47	29.47	gramiketide B ^{13}C
530.39091	Positive	27.47	29.47	gramiketide B ^{13}C X-2
528.38420	Positive	27.47	29.47	gramiketide B ^{13}C X-4
526.37749	Positive	27.47	29.47	gramiketide B ^{13}C X-6
524.37078	Positive	27.47	29.47	gramiketide B ^{13}C X-8
522.36407	Positive	27.47	29.47	gramiketide B ^{13}C X-10
520.35736	Positive	27.47	29.47	gramiketide B ^{13}C X-12
518.35065	Positive	27.47	29.47	gramiketide B ^{13}C X-14

"X" refers to the monoisotopic isotopolog, "X-n" specifies the number (n) of carbon atoms originating from thetracer.

Table S4. Gene annotation of *PKS15* gene cluster (Cluster ID C16) [1].

Position	Gene Code	Description
1	FGSG_04596	related to O-methyltransferase
2	FGSG_04595	related to hydroxylase
3	FGSG_16087	hypothetical protein
4	FGSG_16088	related to 3-ketoacyl-acyl carrier protein reductase
5	FGSG_04593	related to para-hydroxybenzoate polyprenyltransferase precursor
6	FGSG_04592	related to light induced alcohol dehydrogenase Bli-4
7	FGSG_04591	probable farnesyltranstransferase (al-3)
8	FGSG_04590	related to isotrichodermin C-15 hydroxylase (cytochrome P-450 monooxygenase CYP65A1)
9	FGSG_04589	related to tetracenomycin polyketide synthesis O-methyltransferase tcmP
10	FGSG_04588	polyketide synthase

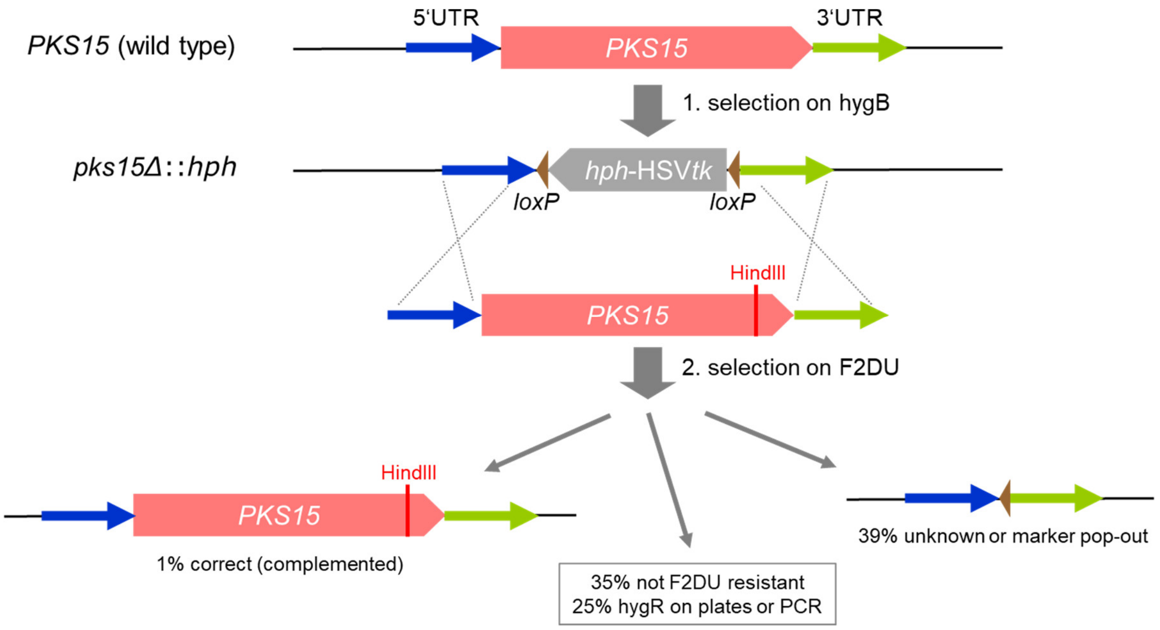

Figure S1. Schematic representation of generation of a *PKS15* in a locus complementation strain. First, the chromosomal *PKS15* gene was replaced by a cassette containing fusion gene allowing positiveselection with hygromycin and negative selection with 5-fluoro-2-deoxyuridine (Herpes simplex thymidine kinase). Recombination with the generated fragment containing upstream and downstreamsequences and the open reading frame of *PKS15* with a silent change creating an additional HindIII sitewas used for recombination. The desired event was found, but most of the resistant transformants hadeither popped out the cassette (recombination at direct *loxP* repeat without Cre recombinase) or were escapes (not resistant upon retesting, still having the hygromycin resistance gene according to PCR).

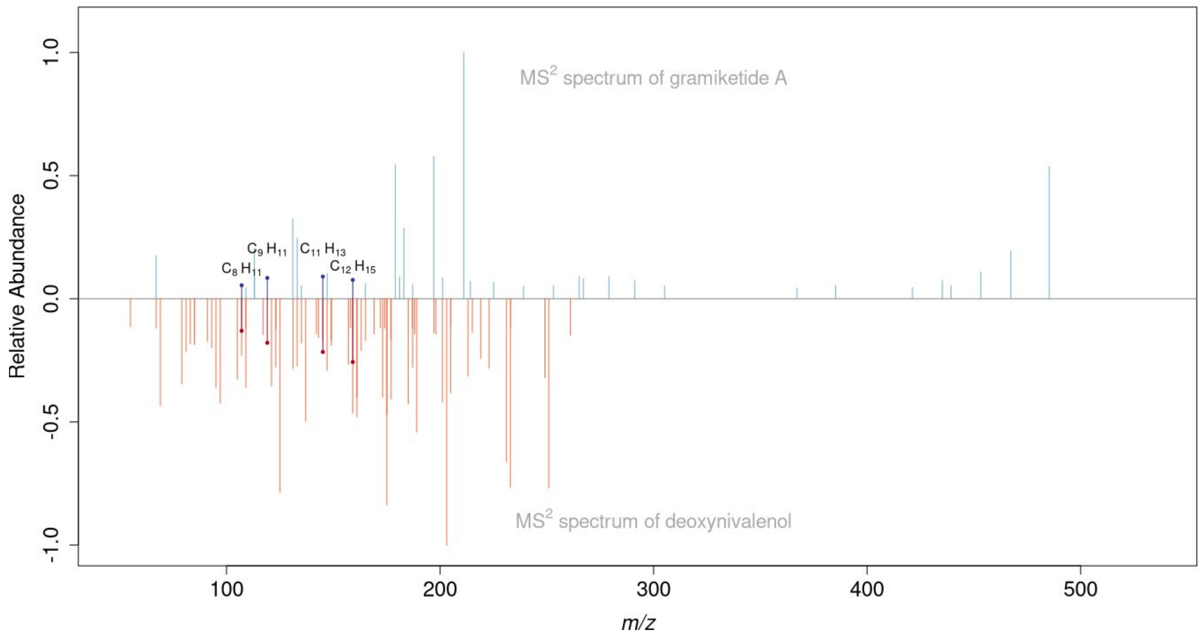

Figure S2. MS/MS spectra comparison of gramiketide A ([M+H]+ ion) and deoxynivalenol ([M+H]+ ion) showing almost no agreement in the fragments (cosine score 0.0024, dot product 0.0126). Only four matching fragments (10 ppm m/z deviation was allowed) of short hydrocarbons were found, namely C8H11, C9H11, C11H13, and C12H15. Due to the low similarity of the spectra,it can be assumed that the structure of the two gramiketide differs significantly from that of deoxynivalenol.

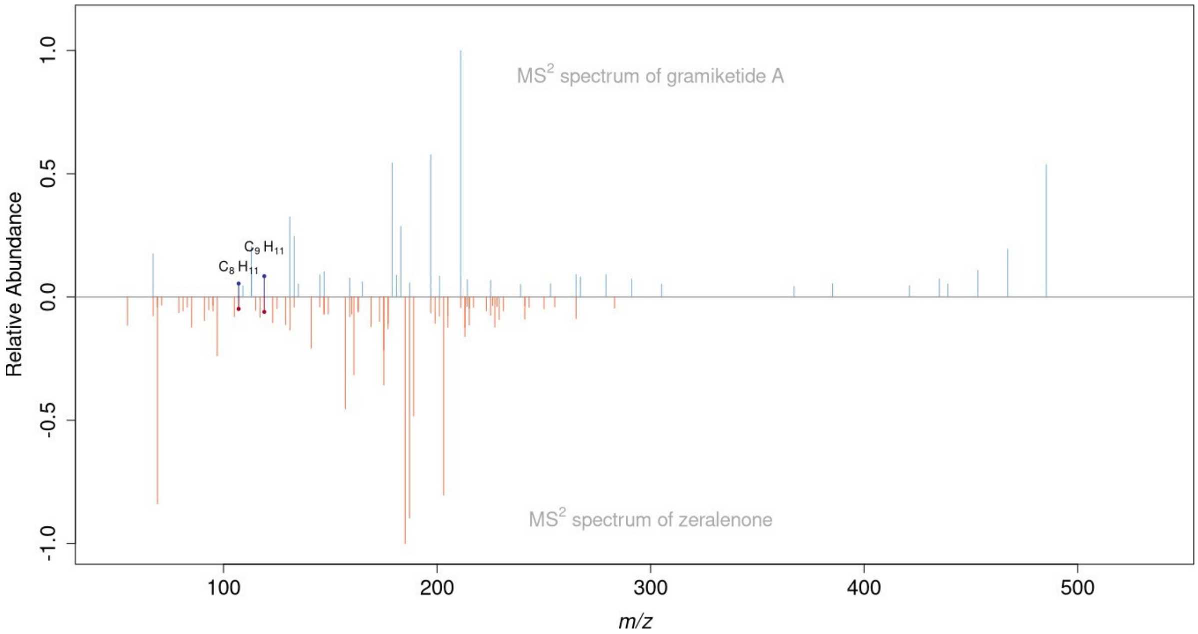

Figure S3. MS/MS spectra comparison of gramiketide A ([M+H]+ ion) and zearalenone ([M+H]+ ion) showing almost no agreement in the fragments (cosine score 0.0157, dot product 0.0024). Only two matching fragments (10 ppm m/z deviation was allowed) of short hydrocarbonswere found, namely C_8H_{11} and C_9H_{11}, which also appeared in the spectra comparison with deoxynivalenol and do not represent an indication of a structural similarity.

References

1. Sieber, C.M.K.; Lee, W.; Wong, P.; Münsterkötter, M.; Mewes, H. -W.; Schmeitzl, C.;Varga, E.; Berthiller, F.; Adam, G.; Güldener, U. The Fusarium graminearum GenomeReveals More Secondary Metabolite Gene Clusters and Hints of Horizontal Gene Transfer. *PLOS ONE* **2014**, *9*, e110311, doi:10.1371/journal.pone.0110311.
